# Functional Performance and Associations between Performance Tests and Neurological Assessment Differ in Men and Women with Parkinson's Disease

**DOI:** 10.1155/2015/519801

**Published:** 2015-10-26

**Authors:** Kadri Medijainen, Mati Pääsuke, Aet Lukmann, Pille Taba

**Affiliations:** ^1^Institute of Exercise Biology and Physiotherapy, University of Tartu, Ülikooli 18, 50090 Tartu, Estonia; ^2^Department of Sports Medicine and Rehabilitation, University of Tartu, Ülikooli 18, 50090 Tartu, Estonia; ^3^Department of Neurology and Neurosurgery, University of Tartu, Ülikooli 18, 50090 Tartu, Estonia

## Abstract

*Background*. Neurological assessment of a patient with Parkinson's disease (PD) is expected to reflect upon functional performance. As women are known to report more limitations even for same observed functional performance level, present study was designed to examine whether associations between neurological assessments and functional performance differ across genders. *Methods*. 14 men and 14 women with PD participated. Functional performance was assessed by measuring walking speeds on 10-meter walk test (10MWT) and by performing timed-up-and-go-test (TUG). Neurological assessment included Hoehn and Yahr Scale (HY), Movement Disorders Society Unified Parkinson's Disease Rating Scale (MDS-UPDRS), Schwab and England Activities of Daily Living Scale (S-E), and Mini Mental State Examination (MMSE). *Results*. In women with PD, Kendall's tau-b correlation analyses revealed significant correlations between functional performance tests and neurological assessment measures, with the exception in MMSE. No corresponding associations were found for men, although they demonstrated better functional performance, as expected. *Conclusion*. Men in similar clinical stage of the PD perform better on functional tests than women. Disease severity reflects upon functional performance differently in men and women with PD. Results indicate that when interpreting the assessment results of both functional performance and neurological assessment tests, the gender of the patient should be taken into consideration.

## 1. Introduction

Parkinson's disease (PD) is a neurodegenerative disease diagnosed mainly based on clinical features. Several gender differences have been reported in symptomatology of PD. According to Haaxma et al. [1], women are older at the onset of the disease and are subject to tremor more often. Women experience nonmotor symptoms such as nervousness, sadness, depression, and constipation more often, whereas men suffer more from daytime sleepiness, drooling, and sex-related symptoms [2]. The information about symptoms is obtained in the form of a patient interview, questionnaires, and objective neurological assessments, which to date are generally not interpreted in a gender-specific context.

There is limited evidence on gender differences in motor performance of PD patients. Hass et al. [3] reported that male participants walk significantly faster, produce larger steps and stride lengths, have a faster cadence, and spend a greater percent of the gait cycle in swing-phase than women. According to Solla et al. [4], women with PD have a higher UPDRS instability score, whereas some studies have reported higher incidence of balance disturbances (e.g., on gait) in men with PD. Augustine et al. [5] found no differences between men and women in motor symptoms or in daily living.

To date, previous research in this field has overlooked the associations between clinical neurological assessment scales and functional performance. For a clinician working with patients with PD, it is important to know whether neurological assessment results are linked to functional performance. Patients with more advanced stages of the disease are expected to perform worse in physical performance tests. This is compliant with the study of Hass et al. [3] which showed that patients with more pronounced Parkinson's disability walked significantly slower. The gait speed of normal walking correlated highly with disease severity also in the study of Hausdorff et al. [6].

Physical tests are less influenced by cultural and educational background and are more beneficial in aspects of validity and reproducibility compared to indirect assessments [7]. Therefore, usage of functional performance tests in both a clinical setting and research could be advantageous. An extensive amount of studies that have included physical performance tests in their methodology has been published in recent years.

To our knowledge, the current research has not looked into the possibility that the associations between functional performance and neurological assessment might be gender specific in patients with PD. However, the population-based study of Rodrigues-Barbosa and colleagues [7] revealed that men (and younger people) had better physical performance. In a study by Baba et al. [8] women with PD demonstrated significantly worse Activities of Daily Living (ADL) capacity. Murtagh and Hubert [9] found also that reporting limitations, usage of assistance, and a greater degree of disability is more probable in women. Older women have been suggested [10] to be more likely to report a higher level of ADL limitation for the same level of observed physical performance compared to men. Buchmann et al. [11] reported men to have greater muscle bulk at all ages, also when looking at participants with a clinical diagnosis of PD.

The main aim of this paper was to evaluate whether the relationships between performance tests and neurological assessment measures differ in women and men with PD. As the neurological and functional assessment results of PD patients are rarely interpreted in gender-specific context, investigating possible sex differences in functional performance and differences in associations among neurological and performance tests were also of interest. Consequently, we hypothesize that male patients with PD have better functional performance and associations between neurological assessments and functional performance differ across genders in patients with PD. The latter might lead to false interpretations about functioning of the patient, which is reasoning behind our study design.

## 2. Materials and Methods

### 2.1. Subjects

Participants were randomly selected from the Estonian Parkinson Disease Epidemiology Database and were diagnosed according to the Queen Square Brain Bank (QSBB) criteria [12]. Inclusion criteria implicated the following: age under 80; disease severity according to modified HY stages 1.5–3.0; absence of dementia (MMSE score of 24 or higher); adequate vision and hearing. Patients with severe dyskinesia and long “off” periods, other neurological problems, acute medical problems, and conditions affecting mobility were excluded. The study was approved by the Ethics Committee of University of Tartu. An informed consent declaration was signed by all participants.

### 2.2. Clinical Assessment

Clinical assessment and measurements of functional performance were conducted on all participants. All assessments were performed while patients were receiving their usual medication and were in “on” state. Clinical evaluation included collecting demographic data, history of the disease, and information on current medications. Neurological assessment comprised HY, MDS-UPDRS, S-E, and MMSE and was performed by a movement disorder specialist. The means and standard deviations for each variable are presented in Table 1 for both genders.

HY is a simple clinical rating scale of PD that defines the motor impairment of patients with PD and is widely used for staging the disease [13]. MDS-UPDRS is a neurological assessment measure created to assess the manifestations of PD. It has four parts, which monitor the influence of PD on nonmotor and motor experiences of daily living; motor examination and questions on motor complications are also included [14]. The total score and the motor score (sum of items from part III in MDS-UPDRS) were used for data analysis. S-E estimates the ability of an individual to live with a disease relative to complete independence [15]. MMSE is used for tracking changes in cognitive functioning and to screen cognitive impairment [16]. Reliable diagnosis of dementia is found to be provided by a cut-off score of 24 (maximum 30) [17].

### 2.3. Functional Performance Assessment

Measures to assess functional performance included timed-up-and-go-test (TUG) and a 10-meter walk test (10MWT). The assessment was performed by three physiotherapists. Prior to conducting the test trials, each of the tests was explained and demonstrated. Patients were barefoot and no participant required an assistive device during functional testing. Patients' blood pressure was measured prior to and during functional performance tests to insure their safety.

Firstly, the 10MWT was carried out on a walkway 12 meters long and 1 meter wide. To indicate the start and stop line for 10MWT, one meter from both ends of the walkway was marked with a red stripe. Patients were instructed to walk from one red stripe to the other red stripe. The time to pass the intermediate 6 meters was measured to allow for acceleration and deceleration. For data analysis, an average of three trials was calculated.

Performing miscellaneous activities requires the ability to adapt walking speed, therefore we included in our study measurement of walking speed at three different speeds: comfortable, maximum, and fast motivated walking speed using motivational instruction. It has been shown that as a person ages the maximum gait speed declines more than the comfortable gait speed [18].

First, the comfortable walking speed was assessed. The test instruction to the participant was walk as he/she would normally walk. Next, the patient was instructed to perform three trials to walk as fast as possible, in order to measure maximum walking speed. Finally, fast motivated gait was measured, by instructing the patient to walk as fast as possible not to miss an imaginary bus in the end of the walkway. This additional motivational instruction was used according to a study by Nascimento et al. [19]. Their study on stroke patients suggested that modified verbal commands or demonstration strategies should be employed by physical therapists to ensure accurate information about maximal gait speed, as the usage of motivational instruction increased the maximal walking speed of the participants.

Secondly, TUG test was used to assess sit-to-stand performance and walking. This test also allows the making of estimations in relation to dynamic balance. The standard protocol of TUG measures the time it takes to stand up, walk a distance of 3 meters, turn, walk back, and sit [20]. The patient was instructed to walk at a comfortable and safe walking speed and the time in seconds was recorded from the command “Go” to the time when the patient was seated again.

Over which shoulder the test is performed is generally left to be decided by the examinee. In present study TUG performance was measured around both shoulders and the average of three trials of the faster performance was used for statistical analysis. In addition to the standard protocol, we included a calculation of walking speed of TUG performance.

### 2.4. Statistics

IBM SPSS 20.0 was used for the database and analyses. Variables across genders were tested for normality with Shapiro-Wilk test. One-way ANOVA was used to compare male and female participants. In addition, partial eta squared was calculated. For each variable Levene's test of homogeneity of variance was also performed. In case of unequal variance the Welch test was used to compare means. Further, the results of functional performance tests were height normalized and same statistical procedures were conducted.

Pearson's coefficient was used to analyze the correlations between functional tests. The relationships between measures of neurological assessments were examined with Spearman rho correlation analysis. Kendall's tau-b correlation analysis was used to assess possible associations between functional performance tests and neurological assessment. Value *p* < 0.05 was considered to be statistically significant.

## 3. Results and Discussion

### 3.1. Differences in Functional Performance of Men and Women with PD

Gait speed is commonly used in clinical research. Walking speed has been shown to be a predictor of a range of outcomes, including survival [21] and fall risk [22]. In present study, the speed of walking was significantly higher in men with PD at all the test conditions used (Table 2).

At the same time, male and female participants did not differ in means of PD stage, disease severity according to MDS-UPDRS, and cognitive function. The effect size (partial eta squared) of gender was under 1% when looking at the results of neurological assessment tests, except for S-E (7%). Anyhow, the level of independence did not differ in male and female participants as indicated in Table 1. Still, in current literature women have consistently reported poorer health status [23, 24].

Significantly faster walking speed of male participants in our study is in compliance with a study by Samson et al. [25] which revealed that absolute values for walking speed are lower in women than men at all ages. Hass et al. [3] demonstrated also faster walking speeds in men compared to women, but the walking speeds in their study were considerably slower. For example, in their study, men in stages 2–2.5 (according to HY) walked on average with a speed of 1.02 ± 0.02 m/s. In comparison, the average speed of comfortable walking was 1.39 ± 0.25 m/s in the present study. This difference can be attributed to the present study excluding acceleration and deceleration as the walking speed was calculated for the intermediate six meters of the 10MWT (2 meters from either end).

Another explanation for the higher walking speeds found in present study might be the particular walkway used. In addition to the red stripes indicating the start and end line, there were black stripes marking every meter. The center of the walkway was also marked with a line running along the entire course of the walkway.

Possibly, the walkway served as a visual cue for the participants. The effect of visual cueing on gait speed and step length of patients with PD has been demonstrated in a number of publications [26, 27]. It is possible that the visual cues were more effective in increasing the walking speed of male participants. Jiang and Norman [28] showed in their study that using transverse visual lines enables a person with PD to start walking with longer steps and higher velocity. Results were most evident in the length of first and second step.

Step length of men is known to be longer [29] and since the distance used in the present study to assess walking speed was short, the impact of transverse lines increasing step length could have been determining these distinct differences in male and female participants. The possible sex differences in effects of cueing in patients with PD need to be examined and verified with further studies.

Differences in walking speed can to some extent be attributed to sex differences in anthropometry. It is well known that on average men are taller [30] and also have longer lower extremities. It is clear that the distance covered in a time unit should be longer for a taller person. It has been demonstrated that a high proportion of the variance in walking speed is accounted for by height in both men and women [31]. Therefore, we compared walking speeds also when walking speeds were normalized for height. Movement speed remained to be higher in men, except for comfortable walking speed. This result is similar to one of our previous works [32], where also no sex differences emerged in walking at a comfortable speed. We hypothesized that, despite lower muscle strength indices, female patients perform relatively better in movements they are more accustomed to.

Walking speed is associated with lower-limb muscle strength [33], which is higher in men [11]. The latter further explains better results of male participants in our study. Anyhow, after height normalization the effect size of gender to the found differences in functional performance tests was under 0.2, indicating a small effect.

The only exception was FWS, which demonstrated a moderate gender effect before (0.394) and strong one after (0.853) height normalization procedure. Possibly, in addition to previously pointed facts, this result could be associated with motivational aspects. Men are known to be more motivated to participate in sporting activities [34]. It can be assumed that male participants might have had higher motivation to physically strain themselves during 10MWT as the maximal walking speed was measured.

The latter assumption is confirmed when looking at the results describing to what extent the participants were able to increase their walking speed compared to comfortable walking speed. On 10MWT, the walking speed of men was significantly higher compared to comfortable walking speed when looking at FWS (*p* = 0.003) and FMWS (*p* = 0.007). On average, the maximal walking speed was 50% faster than comfortable walking speed in men and 20.4% in women. Compared to comfortable walking speed, the fast motivated walking speed was 67.2% faster in men and 41.9% in women. It is noteworthy that considerable variability was found in the ability to increase walking speed from customary to maximal in both men and women.

### 3.2. Differences in Associations between Functional Performance Test and Neurological Assessment of Women and Men with PD

The main aim of this study was to find out whether the relationships between performance tests and neurological assessment measures differ in women and men with PD. The results to answer the main study question are summarized in Table 3.

Results distinctly demonstrate that women with more advanced PD perform worse in functional tests. As PD is a progressive disorder [35], long considered to be a disease primarily causing motor disability [36], these results were expected. Altogether, functional performance is most convincingly associated with S-E and MDS-UPDRS motor examination score, which demonstrated significant associations with all the performance tests in women. As the motor examination part is an examiner rating of the motor manifestations of PD and S-E scale is used to provide an estimation of the patient's ability to function, rated by interviewing the patient [37], finding a relationship with performance tests is a likely outcome.

Comfortable walking speed demonstrated weaker correlation with neurological assessment measures than other performance tests, the associations being significant only with UPDRS-MOT and S-E. The latter supports the hypothesis from one of our previous works [32]: women with PD perform relatively better in movements they are more accustomed to. This aspect might be important to consider when interpreting test results. However, further investigation is needed to verify this aspect.

Explicit differences in associations between neurological and performance tests were detected in men compared to women: in our study, widely used MDS-UPDRS scores, HY, and S-E did not correlate with actual functional performance in men with PD.

We provide some potential explanations for our results. The first relates to the possibility that the test environment was perceived as competitive by men since participants were asked to perform as fast as they possibly could (with due regard for safety), while timing with a stopwatch was openly conducted.

Men tend to be more competitive about sports than women [38]. Deaner et al. [39] also reported that there is substantial sex difference in sports interest. Shekhar and Devi also found differences in achievement motivation of men and women [40]. In a study of Godin and Shephard [41] with older individuals, men showed higher perceived physical self. Consequently, this can result in men being more motivated to strain themselves physically to a greater extent than women. This assumption is supported by found significant gender differences in improving walking speed compared to customary walking speed.

Another explanation for different relationships across genders could be a possibly higher withdrawal of women during performance. Ennis et al. [42] demonstrated that because older adults perceived cognitive efforts as comparatively harder, the level of withdrawal was significantly higher than for younger participants. Withdrawal at harder effort might also apply to physical effort and not be age specific. As men walked faster, FWS testing could have been less difficult for them and as a result men would have strained themselves to a greater extent, whereas women might have withdrawn.

Another possible explanation for sex differences in results might be caused by differences in physical activity. Older women are known to be less active and more sedentary [43]. The latter has been proved also for older adults with chronic diseases [44]. In the present study, eight men and seven women considered themselves to be physically active. Detailed information about physical activity was not collected; therefore the effect of potential differences in physical activity cannot be excluded.

Our results conflict with the previous results by Qutubuddin et al. [45] who found that lower scores on the Berg Balance Scale correlated with higher scores on UPDRS motor score in men with PD. At least to some extent this difference can be attributed to conceptually different assessment methods. The Berg Balance Scale rates balance and consists of 14 items. Each of these items is scored from 0 to 4 and they are added together for a total score between 0 and 56, with a higher score indicating better balance [46]. Although the Berg Balance Scale has been proved to be a reliable assessment measure of functional balance in community-dwelling older adults [47], assessing performance by scoring remains always somewhat subjective compared to objective registration of performance characteristics (duration, speed, strength, etc.).

We found no associations between MMSE and functional performance tests. Due to the relatively small sample size of our study and selection of patients with MMSE scores >24, the generalizations about the effect of cognition on functional performance cannot be made.

In addition to looking for associations between neurological and functional performance tests, we also analyzed the relationships within each: women, who were rated to be in more advanced stage of the disease, also received higher motor and total scores on MDS-UPDRS and had a lower level of independence according to S-E.

Similarly to female participants, MDS-UPDRS total score was associated with estimation of independence in men. On the other hand, in present study stage of PD according to HY was not associated with the other measure used to assess disease severity, MDS-UPDRS in men. Motor examination scores of MDS-UPDRS were associated with neither HY, S-E, nor MMSE in male participants.

Whilst no associations between neurological assessments and functional tests were found in men, the associations found among functional tests were also less clear for male participants than for their female counterparts. For example, a female participant, who walked faster at comfortable speed, also performed faster in the other functional tests (correlations were strong, correlation coefficient *r* being higher than 0.835 at all cases). For male participant CWS demonstrated significant associations only with TUG performance (*r* = 0.537).

The main limitation of our study was the relatively small study sample which limits the usage of several statistical methods, therefore hindering the possible generalizations of our results. However, our results indicate that the gender of the patient with PD influences functional performance and the associations between assessment measures differ across genders. Therefore, when interpreting assessment results, considering sex differences is important for both clinicians and researchers working with patients with PD.

## 4. Conclusions

Men with a similar clinical stage of PD perform better in functional performance tests than women. Functional performance is associated with neurological assessments in women, whereas it is not in men with PD. We recommend considering both neurological and functional assessment measures in a gender-specific context.

Possible sex differences in effects of visual cueing on patients with PD need further investigation.

## Figures and Tables

**Table 1:** General clinical and demographic characteristics of PD patients, subdivided for gender.

Variable	Total PD patients (*n* = 28)	Male PD patients (*n* = 14)	Female PD patients (*n* = 14)	*p* value
Age, years (SD)	70.1 (5.7)	68.2 (6.4)	71.9 (4.4)	0.085
Age at onset, years (SD)	61.5 (7.4)	60.3 (8.5)	62.8 (6.1)	0.379
Disease duration, years (SD)	8.7 (5.5)	8.5 (6.2)	8.9 (5.0)	0.395
HY stage (SD)	2.3 (0.5)	2.3 (0.5)	2.3 (0.6)	0.676
MDS-UPDRS total score (SD)	62.4 (19.6)	58.5 (10.7)	66.4 (25.5)	0.303
MDS-UPDRS motor score (SD)	38.6 (13.8)	37.8 (6.9)	39.4 (18.5)	0.760
S-E %	81.3 (7.4)	83.2 (4.6)	79.3 (9.2)	0.165
MMSE	27.2 (2.0)	27.4 (2.1)	27.1 (1.9)	0.703
Height, cm (SD)	167.3 (10.4)	175.7 (5.6)	157.9 (5.4)	0.000^*∗*^
Body weight, kg (SD)	77.2 (15.1)	85.7 (10.3)	68.6 (14.5)	0.001^*∗*^

PD: Parkinson's disease; *n*: number of patients; *p* value: statistical significance probability of *t*-test comparing men and women; SD: standard deviation; HY: Hoehn and Yahr stage; MDS-UPDRS: Movement Disorders Society Unified Parkinson's Disease Rating Scale; S-E: Schwab and England Activities of Daily Living Scale; MMSE: Mini Mental State Examination; cm: centimeter; kg: kilogram.

**Table 2:** Motor performance of PD patients, by gender.

Variable	Total PD patients (*n* = 28)	Male PD patients (*n* = 14)	Female PD patients (*n* = 14)	*p* value
TUG time, s (SD)	8.56 (3.80)	6.89 (1.40)	10.22 (4.7)	0.023^*∗*^
TUG velocity, m/s (SD)	0.78 (0.21)	0.90 (0.16)	0.66 (0.19)	0.001^*∗*^
CWS, m/s (SD)	1.27 (0.30)	1.39 (0.25)	1.15 (0.30)	0.030^*∗*^
FWS, m/s (SD)	1.73 (0.54)	2.06 (0.42)	1.40 (0.43)	0.000^*∗*^
FMWS, m/s (SD)	1.97 (0.54)	2.29 (0.35)	1.65 (0.52)	0.001^*∗*^
%CWS_FWS (SD)	35.2 (27.1)	50.0 (29.0)	20.4 (14.3)	0.003^*∗*^
%CWS_FMWS (SD)	54.6 (25.5)	67.2 (27.1)	41.9 (16.5)	0.007^*∗*^

PD: Parkinson's disease; *n*: number of patients; *p* value: statistical significance probability; TUG time: the average duration of timed-up-and-go-test; s: second; SD: standard deviation; TUG velocity: the velocity calculated based on duration of timed-up-and-go-test; m/s: meters per second; CWS: customary walking speed during 10 m walk test; FWS: fast walking speed during 10 m walk test; FMWS: fast motivated walking speed during 10 m walk test; %CWS_FWS: the percentage of difference in FWS compared to CWS; CWS_FMWS: the percentage of difference in FMWS compared to CWS.

**Table 3:** Associations between assessed variables in women (*n* = 14) and men (*n* = 14) with PD.

	Parameter	UPDRS-MOT	MDS-UPDRS	HY	S-E
W o m e n	TUG	0.552^*∗∗*^	0.492^*∗*^	0.530^*∗*^	0.679^*∗∗*^
	TUG_vel	−0.575^*∗∗*^	−0.469^*∗*^	−0.530^*∗*^	0.653^*∗∗*^
	CWS	−0.420^*∗*^	−0.313	−0.398	−0.474^*∗*^
	FWS	0.464^*∗*^	−0.425^*∗*^	−0.464^*∗*^	0.576^*∗∗*^
	FMWS	−0.530^*∗∗*^	−0.469^*∗*^	0.497^*∗*^	0.602^*∗∗*^

M e n	TUG	−0.068	0.00	0.073	−0.090
	TUG_vel	0.124	−0.101	−0.091	0.225
	CWS	0.101	0.191	0.018	0.344
	FWS	0.045	0.260	−0.239	−0.241
	FMWS	0.169	−0.056	−0.347	0.225

PD: Parkinson's disease; *n*: number of patients; UPDRS-MOT: motor examination score of MDS-UPDRS; MDS-UPDRS: Movement Disorders Society Unified Parkinson's Disease Rating Scale; HY: Hoehn and Yahr stage; S-E: Schwab and England Activities of Daily Living Scale; TUG: the average duration of “timed-up-and-go-test”; TUG_vel: the velocity calculated based on duration of “timed-up-and-go-test”; CWS: customary walking speed during 10 m walk test; FWS: fast walking speed during 10 m walk test; FMWS: fast motivated walking speed during 10 m walk test; ^*∗*^correlation is significant at the 0.05 level (2-tailed); ^*∗∗*^correlation is significant at the 0.01 level (2-tailed).
